# LY75 Suppression in Mesenchymal Epithelial Ovarian Cancer Cells Generates a Stable Hybrid EOC Cellular Phenotype, Associated with Enhanced Tumor Initiation, Spreading and Resistance to Treatment in Orthotopic Xenograft Mouse Model

**DOI:** 10.3390/ijms21144992

**Published:** 2020-07-15

**Authors:** Sadia Mehdi, Elizabeth Macdonald, Kristianne Galpin, David A. Landry, Galaxia Rodriguez, Barbara Vanderhyden, Dimcho Bachvarov

**Affiliations:** 1 Department of Molecular Medicine, Université Laval, Québec City, QC G1V 0A6, Canada; sadia.mehdi.1@ulaval.ca; 2 Centre de recherche du CHU de Québec, L’Hôtel-Dieu de Québec, Québec City, QC G1X 3S3, Canada; 3 Department of Cellular and Molecular Medicine, University of Ottawa, Ottawa, ON K1N 6N5, Canada; ElMacdonald@ohri.ca (E.M.); kgalpin@ohri.ca (K.G.); davlandry@ohri.ca (D.A.L.); garodriguez@ohri.ca (G.R.); bvanderhyden@ohri.ca (B.V.)

**Keywords:** epithelial ovarian cancer, Ly75, epithelial–mesenchymal transition (EMT), orthotopic xenograft mouse model, hybrid cellular phenotype, cancer stem cells (CSCs), Wnt/β catenin pathway

## Abstract

The implications of the epithelial–mesenchymal transition (EMT) mechanisms in the initiation and progression of epithelial ovarian cancer (EOC) remain poorly understood. We have previously shown that suppression of the antigen receptor LY75 directs mesenchymal–epithelial transition (MET) in EOC cell lines with the mesenchymal phenotype, associated with the loss of Wnt/β-catenin signaling activity. In the present study, we used the LY75-mediated modulation of EMT in EOC cells as a model in order to investigate *in vivo* the specific role of EOC cells, with an epithelial (E), mesenchymal (M) or mixed epithelial plus mesenchymal (E+M) phenotype, in EOC initiation, dissemination and treatment response, following intra-bursal (IB) injections of SKOV3-M (control), SKOV3-E (Ly75KD) and a mixed population of SKOV3-E+M cells, into severe combined immunodeficiency (SCID) mice. We found that the IB-injected SKOV3-E cells displayed considerably higher metastatic potential and resistance to treatment as compared to the SKOV3-M cells, due to the acquisition of a Ly75KD-mediated hybrid phenotype and stemness characteristics. We also confirmed *in vivo* that the LY75 depletion directs suppression of the Wnt/β-catenin pathway in EOC cells, suggestive of a protective role of this pathway in EOC etiology. Moreover, our data raise concerns regarding the use of LY75-targeted vaccines for dendritic-cell EOC immunotherapy, due to the possible occurrence of undesirable side effects.

## 1. Introduction

Epithelial ovarian cancer (EOC) is a disease that is responsible for more cancer deaths among women in the Western world than all other gynecologic malignancies [1]. EOC lethality primarily stems from the inability to detect the disease at an early, organ-confined stage, and the lack of effective therapies for advanced-stage disease. Approximately 70% of patients with advanced-stage EOC have widespread intraperitoneal metastases, including the formation of malignant serous effusions within the peritoneal cavity. Unlike the majority of solid tumors, particularly at the primary site, cancer cells in effusions are not amenable to surgical removal, and failure in their eradication is one of the main causes of treatment failure. Moreover, although 40% to 60% of advanced patients display a complete response rate to standard EOC taxane/platinum-based chemotherapy, most of them relapse after a median period of 18 months due to the emergence of chemoresistance, and only 30% survive 5 years after initial diagnosis [2]. Thus, management of the metastatic disease becomes a crucial problem for the treatment of EOC, and focused identification of novel pro-metastatic target pathways and molecules could enhance the chances of discovering new and effective EOC therapies.

Metastasis is a complex multistep process in the progression of cancer, causing approximately 90% of all human cancer mortalities [3,4]. To colonize a distant secondary site, cancer cells undergo epithelial-mesenchymal transition (EMT) characterized by the suppression of epithelial markers E-cadherin and EpCAM and by the expression of mesenchymal markers such as Snail1/2, Twist1/2, vimentin and N-cadherin, associated with the acquisition of migratory capacity pivotal for invasion and metastasis [5,6,7]. Cancer cells with a high Snail/E-cadherin ratio possess characteristics of mesenchymal cells and display higher mobility and resistance to treatment [8]. Several signaling pathways, including the TGF-β pathway, the Notch pathway and the Wnt/β-catenin pathway, are involved in EMT. Among these, the activation of the Wnt/β-catenin pathway is shown to be an important regulator of EMT in several cancers [9,10,11] including EOC [12,13,14]. Although EMT is clearly important to tumor progression, it is inconsistent with the observation that metastatic lesions mostly exhibit epithelial phenotypes, thus suggesting that mesenchymal-epithelial transition (MET) is critical to the latter stages of metastasis, allowing the mesenchymal cells to re-differentiate into epithelial structures defining the enhanced colonization of secondary sites [15,16].

However, this EMT/MET model is still debated, since as lately shown, circulating tumor cells can form clusters containing cells with both epithelial/mesenchymal (E/M) or hybrid phenotype that display higher metastatic potential and increased drug resistance compared to either epithelial or mesenchymal cells alone [17,18,19], and that breast and EOC metastases are mainly driven by hybrid (E/M) cells [20,21]. Based on these findings, the term epithelial-mesenchymal plasticity (EMP) has been recently introduced, which encompasses both EMT and MET [22,23]. However, it is not yet understood if cells in a hybrid state form a stable and potentially invasive and metastatic population, or whether these cells pass through this hybrid state temporarily and transiently during EMT. Moreover, recent findings demonstrated that epithelial tumor cells do not need EMT to form metastases at secondary sites, and that EMT rather mediates cancer-cell survival by contributing to chemoresistance, since residual cancers following therapy displayed a mesenchymal phenotype and tumors-initiating features [24,25]. Thus, the role of EMT in the cancerogenesis and disease progression, including EOC progression, is still not well defined.

We have previously shown that the antigen receptor LY75 (also known as DEC205/CD205) can modulate EOC cell phenotype and metastatic potential. Indeed, LY75 depletion directed MET in EOC cell lines with mesenchymal-like phenotype (SKOV3 and TOV112), associated with the induction of the E-cadherin, EpCAM and EMP1 expression and the loss of the N-cadherin, TWIST1, FN1 and SNAIL1 expression [26]. Moreover, re-expression of a shRNA-resistant LY75 gene variant in the LY75 knockdown (KD) SKOV3 clones completely restored the initial mesenchymal phenotype and re-established the SKOV3 parental pattern of mesenchymal markers’ expression [26]. We have also demonstrated that the LY75 kD-mediated MET in EOC cells is associated with DNA methylation alterations and suppression of the Wnt/β-catenin pathway [27].

In the present study, we used the LY75-mediated modulation of EMT in EOC cells as a model to investigate *in vivo* the specific implications of EOC cells with epithelial (E), mesenchymal (M) or mixed epithelial plus mesenchymal (E+M) phenotype in EOC initiation, dissemination and treatment response, due to the conflicting data about the roles of these different cellular phenotypes in the EMT-mediated EOC etiology. The disease initiation, spreading and occurrence of therapy resistance was monitored in orthotopic xenograft mouse EOC model, following intra-bursal (IB) injections of SKOV3-M (control), SKOV3-E (Ly75KD) and a mixed population of SKOV3-E+M cells. The IB orthotopic EOC model was chosen due to its advantages over conventional xenograft models (e.g. subcutaneous or intraperitoneal injections of tumor cells), since it reproduces the primary site of tumor formation and enables tumor cells to interact with appropriate microenvironment. Moreover, this model represents quite accurately clinical cancer with regard to common sites of metastases and drug sensitivity [28,29].

## 2. Results

### 2.1. Ly75KD SKOV3 Cells with Epithelial Phenotype (SKOV3-E) Display Enhanced EOC Initiation, Spread, and Resistance to Treatment in Severe Combined Immunodeficiency (SCID) Mice

For our *in vivo* experiments, we used the orthotopic intrabursal (IB) mice model to investigate the tumor-initiating and metastatic potential of EOC cells with mesenchymal (M), epithelial (E) and mixed E+M phenotype. The previously generated cell clones sh-control-SKOV3 (SKOV3-M) and sh-LY75KD-SKOV3 (SKOV3-E) [27] were initially transfected with firefly luciferase plasmid in order to facilitate further monitoring by bioluminescent imaging for tumor formation and spreading. In our initial set (phase 1) of experiments, stable luciferase-expressing SKOV3-M and SKOV3-E clones, as well as a mixed population of SKOV3-E+M cells (1:1 ratio), were directly injected under the bursal membrane (between the bursa and the ovary) of female SCID mice (n = 5 for each experimental group; see Materials and Methods for details).

**Figure 1:**
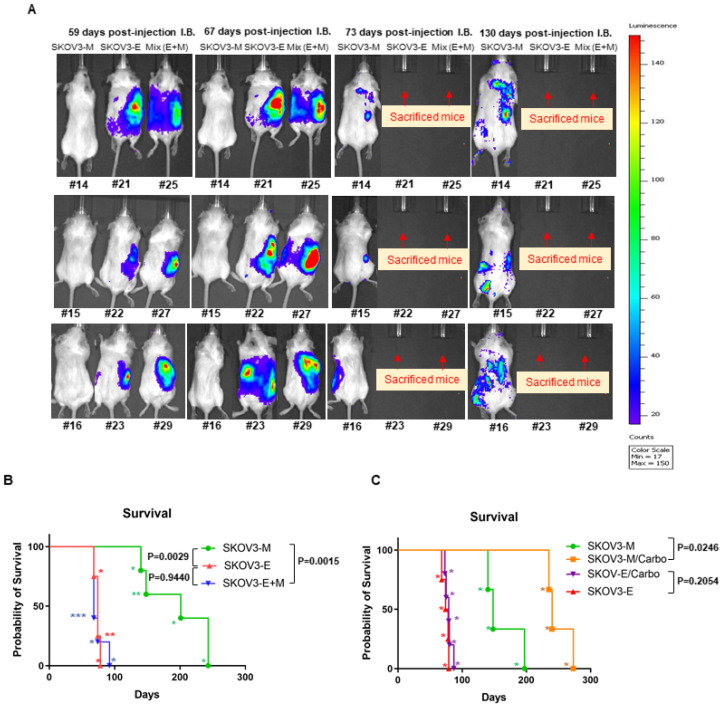
*In vivo* examination of tumor initiation, spreading, survival and response to treatment in SCID mice IB-injected with SKOV3-M SKOV3-E and SKOV3-E+M cells. (**A**) Whole body representative bioluminescence images of the emerging tumors and metastatic lesions in SCID mice 59 days, 67 days, 73 days and 130 days post-injection IB of SKOV3-M, SKOV3-E and SKOV3-E+M cells. (**B**) Survival plots for phase 1 mice IB-injected with SKOV3-M, SKOV3-E and Mix cells. (**C**) Survival plots for phase 2 mice IB-injected with SKOV3-M and SKOV3-E cells, followed by treatment with carboplatin. Asterisks represent the number of sacrificed mice at each step according to the survival time, as indicated in Table 1; see also Section 4.3.5 for details.

We found that the median survival of mice injected with the SKOV3-M cells was 201 days, as primary tumors appeared approximately 73 days post-injection (Figure 1 and Table 1). Mice injected with SKOV3-E and SKOV3-E+M cells displayed quite similar, but significantly lower survival rates, when compared to the SKOV3-M injected animals (74 days and 68 days; p = 0.0029 and p =0.0015, respectively), as primary tumors appeared approximately 55 days post-injection (Figure 1 and Table 1).

In addition to the observed primary tumors, all SKOV3 phenotypes (M, E and E+M) generated metastases in the intraperitoneal space, affecting the diaphragm, liver, pancreas and omentum. However, we noticed additional dorsal metastases in mice injected with SKOV3-E and SKOV3-E+M cells. Detailed data on the survival values of each experimental animal, as well as the medium survival rates and tumor spreading (metastatic) sites of the three experimental groups, are presented in Table 1.

We further carried additional (phase 2) experiments in order to evaluate the treatment’s effect on disease progression in mice injected with different SKOV3 cellular phenotypes. Since, in our phase 1 experiments, mice injected with SKOV3-E or SKOV3-E+M cells displayed almost identical disease initiation, spreading and survival rates, we further evaluated the response to carboplatin treatment of four additional groups of experimental animals (n = 5) previously IB-injected with either SKOV3-E or SKOV3-M cells.

For two of the experimental groups, injected with SKOV3-E or SKOV3-M cells, intraperitoneal injection of carboplatin was weekly applied at a dose of 60 mg/kg body weight over a 3-week period, while the other two groups, injected with SKOV3-E or SKOV3-M cells respectively, were used as controls (placebo injected). The carboplatin treatment started 73 days post-injection for the SKOV3-M treated mice, and 55 days post-injection for the SKOV3-E treated mice, based on the phase 1 data of the initial appearance of the primary tumors in these experimental animal groups. As shown on Figure 1C, SKOV3-E-injected mice displayed essentially identical survival rates, as seen in our phase 1 experiments (see Figure 1B for comparison), with practically no difference between the carboplatin-treated and non-treated (control) animals. Similarly, SKOV3-M-injected mice from the control group showed similar survival rates as those found in our phase 1 experiments; however, carboplatin-treated animals from the SKOV3-M treated group displayed a significantly longer survival (*p* = 0.0246) than those from the corresponding control group (Figure 1C).

**Figure 2:**
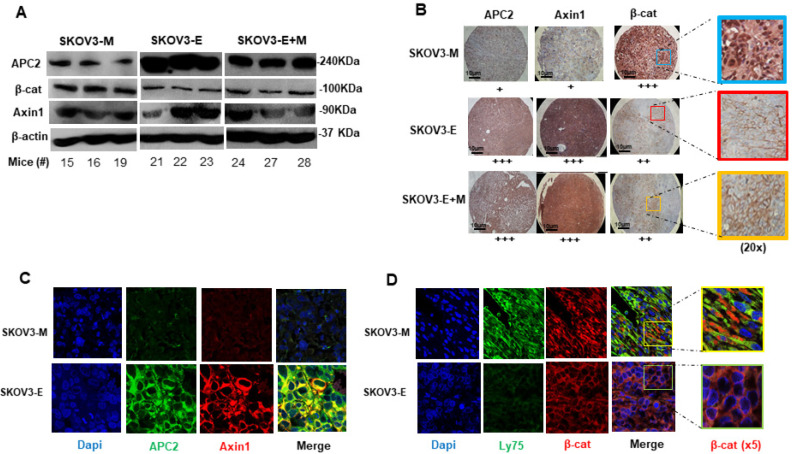
Analysis of the Wnt/B-catenin pathway status in primary tumor tissue samples of phase 1 mice experimental groups IB-injected with SKOV3-M, SKOV3-E or SKOV3-E+M cells. (**A**) Western blot analysis of β-catenin, APC2 and Axin1 proteins in SKOV3-M (mice 15, 16 and 19), SKOV3-E (mice 21, 22 and 23) and SKOV3-E+M mice (samples 24, 27 and 28). β-actin was used as the loading control. (**B**) Immunohistochemistry (IHC) analysis of the expression of β-catenin, APC2 and Axin1 and β-catenin sub-cellular localization in tumor tissues extracted from mice injected with the SKOV3-M, SKOV3-E and SKOV3-E+M cells at 40× (scale bar 10 μm). The plus symbols (+ to +++) indicate the level of expression (from lower to higher) of the corresponding protein. (**C**) Immunofluorescence (IF) analysis of β-catenin, APC2 and Axin1 expression in tumor tissues extracted from mice injected with SKOV3-M and SKOV3-E cells (magnification at 60×). (**D**) IF analysis of Ly75, β-catenin and β-catenin sub-cellular localization in tumor tissues extracted from mice IB-injected with SKOV3-M and SKOV3-E cells (magnification at 40×).

We have previously shown that LY75 supports the active status of the Wnt/β-catenin pathway in EOC cells, and the LY75 depletion results in the suppression of Wnt/β-catenin activity, associated with decreased β-catenin expression and the strong induction of Axin1 and APC2, representing members of the Wnt/β-catenin suppressor complex [27]. Analysis of primary ovarian tumor samples obtained from our phase 1 experimental animals confirmed these data, as tumor tissues derived from SKOV3-E- and SKOV3-E+M-injected mice displayed considerably diminished β-catenin expression and enhanced Axin1 and APC2, following both Western blot and immunohistochemistry (IHC) analyses, as compared to SKOV3-M-injected mice (Figure 2A,B). Moreover, β-catenin, Axin1 and APC2 also exhibited similar protein expression patterns in tumors from SKOV3-E- and SKOV3-M-injected mice following immunofluorescence (IF) analysis (Figure 2C), as the β-catenin expression profile seemed to be more concentrated onto, and around, the nuclei in SKOV3-M tumor tissues, while β-catenin displayed enriched plasma membrane localization in SKOV3-E and SKOV3-E+M tumors (Figure 2B,D).

### 2.2. SKOV3-E Cells Display Hybrid (E/M) Phenotype and Cancer Stem Cells (CSCs) Features

**Figure 3:**
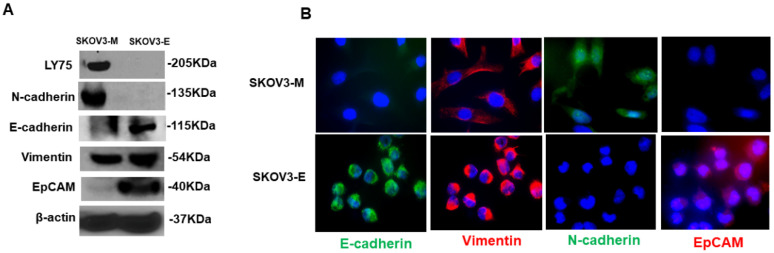
Protein expression analysis of different EMT markers in SKOV3-M and SKOV3-E cells. (**A**) Western blot analysis of the protein expression of LY75, N-cadherin, E-cadherin, vimentin and EpCAM in SKOV3-M and SKOV3-E cells. β-actin was used as a loading control. (**B**) Immunofluorescence analysis of the protein expression of N-cadherin, E-cadherin, vimentin and EpCAM in SKOV3-M and SKOV3-E cells (magnification at 60×).

The above results were more or less unexpected since, as repeatedly shown, cancer cells with a mesenchymal phenotype display higher migration and invasion potential and enhanced resistance to treatment, when compared to cancer cells with epithelial morphology [5,30,31]. This prompted us to characterize the EMT-related phenotypic features of the SKOV3-E cell clones used in our *in vivo* experiments as more profoundly significant. The expression levels of several EMT markers were analyzed in SKOV3-E cells, in comparison to SKOV3-M cells. Western blot analysis of the protein expression levels of the epithelial markers E-cadherin and EpCAM, and the mesenchymal markers N-cadherin and vimentin, confirmed our previous findings [26] concerning the weak (absence of) E-cadherin and EpCAM expression, and the strong N-cadherin and vimentin expression, in SKOV3-M cells, as well as the high levels of E-cadherin and EpCAM expression and weak N-cadherin expression in SKOV3-E cells (Figure 3A). However, even stronger vimentin expression was also found in SKOV3-E cells (Figure 3A), which was consecutively confirmed by IF analysis (Figure 3B).

**Figure 4:**
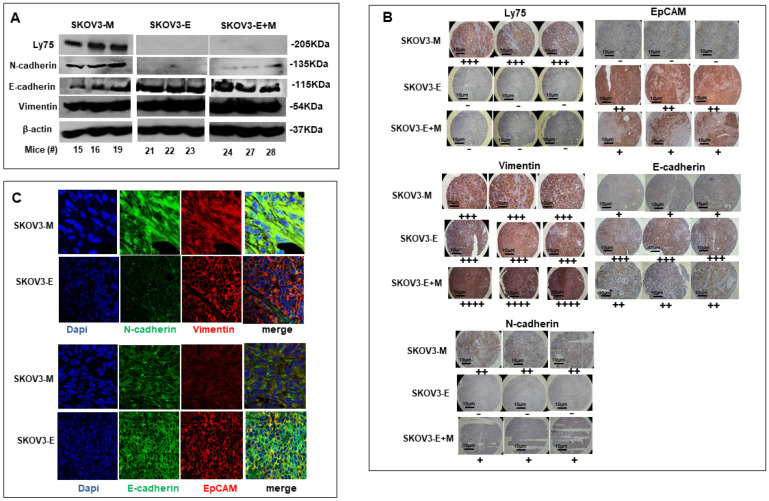
Protein expression analysis of different EMT markers in tumor tissues extracted from mice IB-injected with SKOV3-M, SKOV3-E and SKOV3-E+M cells. (**A**) Western blot analysis of the protein expression of LY75, vimentin, N-cadherin and E-cadherin, and EpCAM in tumor tissues extracted from mice IB-injected with SKOV3-M, SKOV3-E and SKOV3-E+M cells. β-actin was used as a loading control. (**B**) Representative immunohistochemistry (IHC) images of the protein expression of LY75, vimentin, E-cadherin, N-cadherin and EpCAM in tumor tissues extracted from mice injected with the SKOV3-M, SKOV3-E and SKOV3-E+M cells. Magnification at 40× (scale bar 10 μm). The plus symbols (+ to ++++) indicate the level of expression (from lower to higher) of the corresponding protein. The minus (−) symbol means no expression. (**C**) Immunofluorescence analysis of the protein expression of N-cadherin, vimentin, E-cadherin and EpCAM in tumor tissues extracted from mice IB-injected with SKOV3-M and SKOV3-E cells (magnification at 60×).

These data were further supported by Western blot, IHC and IF analyses of the expression levels of these four EMT markers in ovarian tumor samples obtained from our phase 1 experimental animals. As seen from the Western blot presented on Figure 4A, strong vimentin expression was observed in tumor tissues derived from mice injected with SKOV3 cells, comprising all three phenotypes tested (M, E and E+M), while tumors from SKOV3-M-injected animals displayed strong N-cadherin and weak E-cadherin expression, and tumors derived from SKOV3-M and SKOV3-E+M mice showed very high E-cadherin expression and a lack of N-cadherin expression. Further IHC and IF analyses using tumor tissues of phase 1 experimental animals were strongly confirmative for the above-described differential expression patterns of the four EMT markers, including the strong vimentin expression in all experimental groups (Figure 4B,C), suggesting that SKOV3-E cells could exhibit a hybrid phenotype.

**Figure 5:**
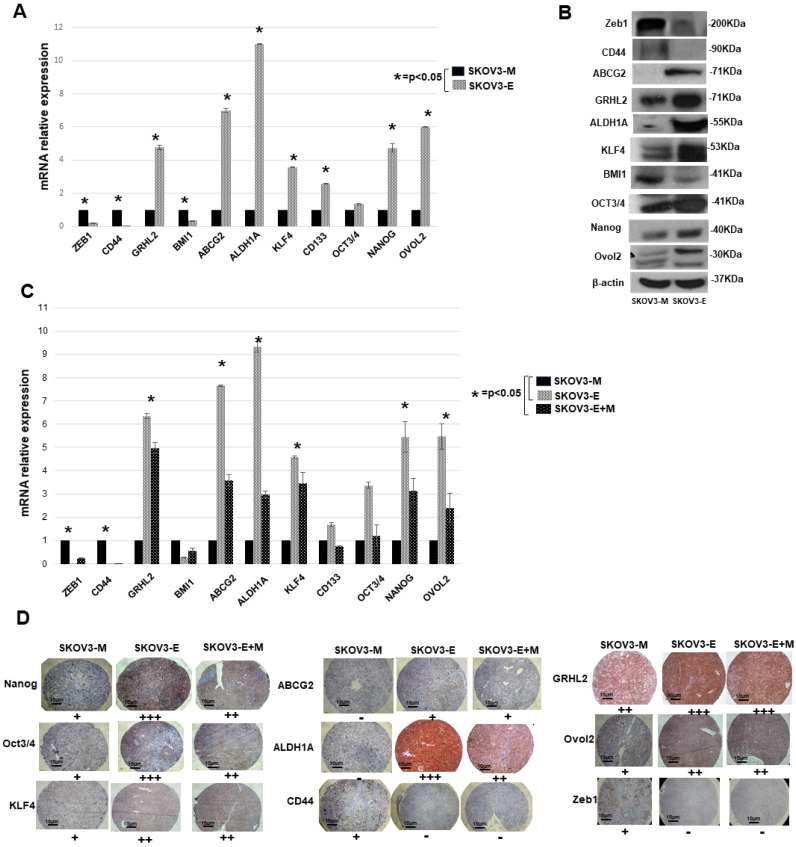
Gene (mRNA) and protein expression levels of different CSCs markers and hybrid state stability factors in SKOV3-M and SKOV3-E cells, and in tumor tissues extracted from mice IB-injected with SKOV3-M, SKOV3-E and SKOV3-E+M cells. (**A**) Quantitative PCR (qPCR) analysis of the mRNA expression levels of some CSCs markers (CD44, BMI1, ABCG2, ALDH1A, KLF4, CD133, OCT3/4 and NANOG) and genes implicated in the control of the hybrid cellular state (ZEB1, GRHL2 and OVOL2), in SKOV3-M and SKOV3-E cells. (**B**) Western blot analysis of the protein expression levels of some CSCs markers (CD44, ABCG2, ALDH1A, KLF4, BMI1, OCT3/4, Nanog) and genes implicated in the control of the hybrid cellular state (ZEB1, GRHL2 and OVOL2), in SKOV3-M and SKOV3-E cells. (**C**) qPCR analysis of the mRNA expression levels of some CSCs markers (CD44, BMI1, ABCG2, ALDH1A, KLF4, CD133, OCT3/4 and NANOG) and genes implicated in the control of the hybrid cellular state (ZEB1, GRHL2 and OVOL2), in tumor tissues extracted from mice IB-injected with SKOV3-M, SKOV3-E and SKOV3-E+M cells. Data are presented as mean ± SD. *, *p* < 0.05; (Student’s t test). (**D**) Representative IHC images of Nanog, Oct3/4, Klf4, ABCG2, ALDH1A, CD44, GRHL2, OVOL2 and EpCAM expression in tumor tissues extracted from mice injected with the SKOV3-M, SKOV3-E and SKOV3-E+M cells. Magnification at 40× (scale bar 10 μm). The plus symbols (+ to +++) indicate the level of expression (from lower to higher) of the corresponding protein. The minus (−) symbol means no expression.

Since cancer cells (including EOC cells) bearing the hybrid E/M phenotype are shown to be associated with increased tumor stemness, and thus to display some CSCs features [20,32,33], we verified the expression of different CSCs markers in both SKOV3-M and SKOV3-E cells. We found that, excluding CD44 and BMI, which are more strongly expressed in SKOV3-M cells, all the other CSCs markers tested (including OCT3/4, KLF4, ABCG2, Nanog, CD133 and ALDH1A) displayed significantly higher mRNA and protein expression levels in the SKOV3-E cells, as compared to the SKOV3-M cells (Figure 5A,B). These data were further confirmed by analyzing the mRNA (qPCR) and protein (IHC) expression levels of most of the CSCs markers listed above in the tumor samples derived from SKOV3-M-, SKOV3-E- and SKOV3-E+M-injected animals (Figure 5C,D).

Finally, the suggested SKOV3-E hybrid phenotype was supported by the strong expression of the hybrid state–associated phenotypic stability factors (PSFs) OVOL2 and GRHL2 in the SKOV3-E cells, and in tumors derived from SKOV3-E- and SKOV3-E+M-injected animals (Figure 5), while the EMT progression factor Zeb1 was more sustained in SKOV3-M cells and tumor samples from the SKOV3-M-injected mice (Figure 5).

## 3. Discussion

The main objective of this study was to investigate the role of EMT in EOC initiation, progression and treatment response *in vivo* in intrabursal (IB) orthotopic xenograft mouse EOC model. More specifically, we used SKOV3 EOC cells with Ly75 expression-modulated E or M phenotype for IB injections in SCID mice, to better understand the specific implication of EOC cells with different EMT-related phenotypes in EOC dissemination. Initially, the results of our *in vivo* experiments were rather surprising, as we were expecting that EOC cells with mesenchymal phenotype (SKOV3-M cells) will display higher spreading, metastatic potential and resistance to treatment, compared to epithelial SKOV3 (SKOV3-E) cells. Moreover, a mixed population (1:1 ratio) of SKOV3-M and SKOV3-E cells (SKOV3E+M cells) displayed quite similar tumor initiating and spreading characteristics, including resistance to treatment when compared to SKOV3-E cells, suggesting that the portion of SKOV3-E cells in this cellular mix is the one responsible for such a high aggressiveness in tumor formation and treatment response. As previously reported in the literature, only mesenchymal EOC cells and mesenchymal EOC cell-containing aggregates were able to invade through 3-dimensional collagen-rich matrices [34], and EMT cells with complete mesenchymal or partial (hybrid) EMT phenotype were much more aggressive in driving ascites formation and development of peritoneal metastases than epithelial EOC cells [20,35]. Indeed, we have previously shown that SKOV3-E (Ly75KD) cells, intraperitoneally (IP) injected in SCID mice, resulted in enhanced tumor cell formation and metastatic growth *in vivo* [26], but this was to be expected, since EOC metastases frequently appear disseminated throughout the peritoneum [36], and metastatic lesions mostly exhibit epithelial phenotypes due to the increased capacity of epithelial cells to colonize distant sites [26].

In cancer, EMT has been initially considered as a binary process with two very distinct cell populations - epithelial and mesenchymal, characterized by the loss of expression of the epithelial marker E-cadherin and the gain of the expression of the mesenchymal markers N-cadherin and vimentin [37]. However, there is accumulating evidence that EMT occurs progressively, and the cells pass by intermediate states displaying transcriptional, morphological and epigenetic E/M (hybrid) features, a process called partial/incomplete EMT, or EMP [38,39,40]. Further studies in various cancers (including EOC) have shown that that hybrid cells can acquire a multipotent stem cell like phenotype associated with higher proliferative and invasive characteristics and resistance to treatment [20,41].

We now show that SKOV3-E cells display hybrid phenotype and stemness characteristics, which could explain their aggressive behavior in our experimental EOC animal model. As reported before [26,27], SKOV3-M cells exhibited a typical EMT mesenchymal markers-expression profile, while SKOV3-E (Ly75KD) cells have displayed EMT-related epithelial features; however, we have now found that SKOV3-E cells also strongly express the mesenchymal marker vimentin (Figure 3), as a strong vimentin expression was also retained in tumors, extracted from mice, IB-injected with SKOV3-E and SKOV3-E+M cells (Figure 4). Vimentin is highly expressed in most epithelial cancers including EOC, and its expression correlates with disease progression and poor prognosis [42,43]. Moreover, CSCs expressing vimentin, NANOG and other markers of pluripotency were found in tumor tissue sections of women with high grade serous ovarian carcinoma [44]. Indeed, we have also shown that SKOV3-E cells express a number of CSCs markers, including EpCAM, ALDH1A, ABCG2, CD133, KLF4, OCT3/4 and NANOG (see Figure 3; Figure 5A,B), as the expression profile of these CSCs markers was preserved in tumors of mice, IB-injected with SKOV3-E cells (Figure 4B,C and Figure 5C,D). All these CSCs markers have been previously shown to be implicated in the maintenance of EOC stem cells, including their aggressive behavior and resistance to treatment [45,46,47,48,49,50,51]. The correlation between the expression of CSCs markers and the development of cancer (initiation, progression, and spreading) is questioned and seems to differ from one cancer type to another. Indeed, each CSCs marker shows independent expression levels and seems to interact with other CSCs markers at different stages of tumorigenesis. Thus, several studies have reported that ALDH1(+)CD133(+)EpCAM(+) cells have the potential to initiate the EOC spread and drug resistance [13,52]. The highly tumorigenic OVCAR5 EOC cell line has been also characterized by the simultaneous expression of the pluripotency-associated genes including CD133, NANOG, OCT4 and KLF4 [53]. When compared to SKOV3-E cells, SKOV3-M cells also exhibited some CSCs characteristics, but most of the above listed pluripotency markers were presented with weaker/loss of expression, as only two CSCs markers - BMI1 and CD44 exhibited enhanced expression in these cells, as well as in tumors extracted from SKOV3-M-injected experimental animals (Figure 5A–C).

Importantly, SKOV3-E cells, as well as tumor tissues of mice IB-injected with SKOV3-E and SKOV3-E+M cells, displayed enhanced expression of OVOL2 and GRHL2 - two phenotypic stability factors (PSFs), previously shown by us and others to stabilize the highly aggressive and metastatic E/M (hybrid) phenotype of cancer cells [17,38,54,55] (see Figure 5). Moreover, these PSFs repress the EMT progression factor Zeb1 to prevent the EMT advancement to a complete mesenchymal state [17,38,55], and our data are indicative for weaker/lack of expression of Zeb1 in SKOV3-E cells and tumor tissues of mice IB-injected with SKOV3-E and SKOV3-E+M cells, compared to SKOV3-M cells and corresponding tumors.

As previously stated [26], our findings raise again concern on the use of LY75-targeted vaccines for dendritic-cell EOC immunotherapy. LY75 is a type I transmembrane surface protein that belongs to the macrophage mannose receptor (MMR) family of C-type lectin endocytic uptake receptors, as the LY75 receptor plays an important role in antigen uptake for presentation and cross-presentation to T cells and the initiation of the immune response [26]. Accordingly, loading of LY75 with antigens is widely used in vaccination including cancer immunotherapy. This approach has been exploited *in vitro* and *in vivo*, both in mice and humans, to deliver specific antigens to dendritic cells, and induces specific CD81 and CD41 T-cell responses [26]. However, and based on our data, blocking the LY75 receptor with specific antibodies could generate undesirable side effects, associated with the acquisition of the more aggressive hybrid phenotype and stemness features in mesenchymal-type tumor cells.

Finally, our *in vivo* data have also confirmed our previous findings [27] for the direct implication of the LY75 receptor in modulating the Wnt/β-catenin signaling in EOC cells. Here, we show that Ly75 gene ablation also directed the suppression of the Wnt/β-catenin pathway in tumor samples from mice IB-injected with SKOV3-E and SKOV3-E+M cells. These findings were initially surprising, since Wnt/β-catenin signaling has been shown to be associated with partial EMT states and the acquisition of stemness traits in cancer cells [56], although some protective functions of the Wnt/β-catenin pathway have been also suggested, as active Wnt/β-catenin signaling was associated with the inhibition of melanoma growth [57]. There is also growing evidence that the Wnt/ β-catenin pathway regulates many key aspects of EOC progression, including the maintenance of CSCs, metastases, and chemoresistance [58,59]; however, the mechanisms underlying the hyperactivation of the Wnt/β-catenin pathway and its role in EOC etiology are not entirely clear [60]. Indeed, data from a large genome-wide association study (GWAS) in EOC were indicative for a significant downregulation of the Wnt pathways-agonist WNT4 in ovarian tumors [61]. Moreover, a recent study has demonstrated that the inactivation of the Wnt/β-catenin pathway is required for maintaining stemness and preventing the differentiation of organoid cultures derived from high-grade serous ovarian cancer (HGSOC) patients, and that HGSOC progresses more aggressively in an environment free of Wnt [62].

In conclusion, we have shown that Ly75 suppression in the mesenchymal EOC cell line SKOV3 generates cells bearing a hybrid (E/M) phenotype and stemness characteristics, associated with a strong expression of the PSFs OVOL2 and GRHL2. These SKOV3-Ly75 KD cells directly enhanced EOC initiation, tumor spread and resistance to treatment following intra-bursal IB injection in severe combined immunodeficiency (SCID) mice, compared to mesenchymal-type SKOV3 cells. As previously suggested [27], we also demonstrated *in vivo* that LY75 supports an active status of the Wnt/β-catenin pathway in EOC cells, while the LY75 depletion predominantly directs the pathway suppression, indicative for a protective role of this pathway in EOC etiology. Moreover, our data raise concern on the use of LY75-targeted vaccines for dendritic-cell EOC immunotherapy due to possible occurrence of undesirable side effects. A comprehensive understanding of molecular processes and the chronology of events triggering EMT in EOC could arrange for the development of more effective treatment strategies for this deadly disease.

## 4. Materials and Methods

### 4.1. Cell Cultures

The SKOV3 cell line is purchased from American Tissue Type Collection (Manassas, VA, USA). Cells were maintained in RPMI-1640 medium supplemented with 10% (v/v) fetal bovine serum (Hyclone, Logan, UT, USA) and cultured in a humidified incubator with 5% CO_2_ at 37°C as previously described [27].

### 4.2. Firefly Luciferase Stable Transfection

SKOV3 clones (SKOV3-M and SKOV3-E) were stably transfected with the firefly luciferase gene plasmid pcDNA3 (#18964, Addgene, Cambridge, MA, USA) using turbofect reagent and according to the manufacture instructions (#R0532, Thermo Fisher Scientific, Ottawa, ON, Canada). Stable cells population were selected by incubation with 800 µg/mL of G418 and then maintained in RPMI medium with 500 µg/mL of G418. 

To test the luciferase activity, one day prior to the experiment 1.5 × 10^4^ SKOV3-luc cells were plated per well in 100 μL of standard RPMI medium, in 96-well plates. The next day, the cells were harvested, and luciferase activity was quantified on a Tristar LB941 Multimode Microplate Reader (by Berthold Technologies GmbH & Co. KG, Bad Wildbad, Germany) using a Luc-Pair™ Firefly Luciferase HS Assay Kit (GeneCopoeia, Rockville, MD, USA). Firefly luciferase activity was then normalized to non-transfected control cells. Clones displaying more stable luciferase signals were further selected for animal injections. 

### 4.3. Animal Experiments

Animal experiments were carried out at the University of Ottawa under a protocol approved on 14th of March 2018 by the Animal Care Committee (protocol approval number OHRIe-2120) and conformed with or exceeded standards outlined in the Canadian Council on Animal Care Standards (CCAC) and the Animals for Research Act, R.S.O. 1990, Chapter c. A.22. Female Fox Chase SCID mice (Charles River; CB17/Icr-Prkdcscid/IcrIcoCrl) were randomly assigned to cages in groups of 4 upon arrival and were acclimated in conventional micro-isolator cages with plastic huts and corn cob bedding for 1 week prior to commencement of the trial. SCID mice were maintained in a dedicated room for immune compromised mice (21 °C, 40–60% humidity, 12/12-h light/dark cycle) with a commercial rodent diet (2018 Teklad Global 18% Protein Rodent Diet, Harlan Laboratories), along with acidified water available ad libitum. Housing, food and water were autoclaved, and animal manipulations were carried out in a certified ESCO type A2 BSC hood, following a two-person dirty/clean protocol. 

#### 4.3.1. Study Design

The experiments were carried out in two phases. All mice were allocated to cages in groups of 5 following intra-bursal surgeries. Cages of phase 1 mice (*n* = 15; 7 weeks old) housed 1 mouse, allocated randomly, from each of the three experimental groups [SKOV3-M, SKOV3-E and SKOV3-E+M, representing a mixed cellular population of SKOV3-M and SKOV3-E cells (50:50)]. Cages of phase 2 mice (*n* = 20; 7 weeks old; average weight 17.3 ± 0.94 g) housed mice from the experimental groups SKOV3-M or SKOV3-E. Wellness assessments were carried out by Animal Care staff daily, and necropsies of end pointed mice were blinded with regard to the treatment group. End of wellness criteria were based on several factors, including signs of pain, Body Condition Score (BCS), respiratory distress, pallor and coat condition. End point mice were euthanized in Ancare static microisolators by CO_2_ asphyxiation delivered at a flow rate of 20–30% chamber volume per minute.

#### 4.3.2. Intra-Bursal Injections

One hour prior to surgery, mice were given subcutaneous (s.c.) buprenorphine HCL (0.1 mg/kg, PHR1729) for pain management. At time of sacrifice, mice were anesthetized (3% isofluorane; Fresenius Kabi, Canada and 1% oxygen), given fluids (1 mL Saline, s.c.) and received an application of eye ointment (Optixcare Eye Lube; Aventix). The dorsal surface of the mouse was shaved and cleaned using antiseptic (SoluPrep; 2% chlorhexidine in 70% alcohol, 3M cat # 7000136295). Once prepped, a 2–3-cm dorsal wall incision was made followed by a 0.5-cm incision in the peritoneal wall. The fat pad enveloping the ovary was exteriorized and held in place using a bulldog clamp. One ovary of each mouse received an injection of cells (2 μL of a 5.0 × 10^4^ cell suspension) using a custom Hamilton syringe (50 μL capacity, supplied with small hub RN needle, 4-point style, 30-guage, 52° angle, 3/4” needle length, Cat#7803-07) and dispenser repeater (Hamilton, Cat# 1482225). The ovary was carefully returned to the peritoneal cavity, which was then closed with proline sutures [6/0 Surgipro, VP-718X, CV-11 (taper)]. The dorsal body wall was stapled (VWR, Cat#CA-BD427631), and transdermal Bupivicane (2%, 0.1 mL/animal BID; Chiron Compounding Pharmacy) was applied over the incision and staples. The entire process required approximately 10 min, during which time mice were maintained at 3% isofluorane/1% oxygen on a sterile gauze-covered heated pad. All mice were maintained and observed for complications while recovering from the anesthetic in a heated incubator box. Upon regaining consciousness, mice were randomized to cages as per the experimental design. Post-operative care included s.c analgesic (Buprenorphine HCL) administration (q8-12; 36–48 h post-op), observation and staple removal 7–10 days later.

#### 4.3.3. *In Vivo* Bioluminescence Imaging

All *in vivo* imaging was carried out on the IVIS^®^ Spectrum *in vivo* imaging system (Caliper Life Sciences, PerkinElmer, Guelph ON, Canada) and images were analyzed with the Living Image^®^ advanced *in vivo* imaging software (Version 4.3.1; PerkinElmer, Guelph ON, Canada). D-Luciferin (Xenolight D-Luciferin Potassium salt; PerkinElmer # 122799) at a dose of 150 mg/kg body weight was delivered intraperitoneal (i.p.) (phase 1 trial) and s.c. (phase 2 trial) using a 25 G × 5/8 needle. At 10 min post-injection, mice were anesthetized with Isoflurane (3% isoflurane/1% oxygen) and received an application of eye ointment. Mice were imaged 15 min post-injection. Anesthetization was maintained throughout imaging with 3% Isoflurane. Image acquisition was carried out on groups of 3 mice (cage mates) at a time. Following image acquisition, mice were returned to their cage and monitored to ensure full recovery from anesthetic. 

#### 4.3.4. Carboplatin Treatment

One group of SKOV3-M (*n* = 5) and one group of SKOV3-E (*n* = 5) phase 2 mice were treated intraperitoneally with carboplatin (DIN 02320371) at a dose of 60 mg/kg body weight (*n* = 10), weekly over a 3-week period. Respectively, one group of SKOV3-M (*n* = 5) and one group of SKOV3-E (*n* = 5) phase 2 mice were treated intraperitoneally with an equal volume of placebo (PBS). All mice were monitored and provided with supportive care in the form of soaked food as needed.

#### 4.3.5. Data Analysis

Of the mice entered in the phase 1 trial, 14 were included in the analysis. These included 5 mice of the SKOV3-M group, 4 mice of SKOV3-E group and 5 mice of the SKOV3-E+M group. One mouse from SKOV3-E group was further excluded due to lack of tumor development. From the phase 2 group, 3 SKOV3-M mice and all 5 SKOV3-E mice treated with carboplatin were included in the analysis. Two SKOV3-M mice died due to anesthetic complications during imaging. Two SKOV3-M (placebo) mice and one SKOV3-E (placebo) mouse were also excluded from the analysis, due to anesthetic complications during imaging or lack of detectable tumor by imaging. 

### 4.4. Statistical Analysis

The Prism software (Version 7.0d, GraphPad Software, San Diego, CA, USA) was used to determine the statistical significance of differences in the means of experimental groups. A Kaplan–Meier plot was used to analyze the survival of animal groups used in the study, with a log-rank test to represent survival significance. A significant association was considered when *p*-values were < 0.05. 

### 4.5. Immunohistochemistry (IHC)

IHC analyses of the expression of different markers in tumor tissues derived from the experimental animals were performed as previously described [26] and all retrieval methods were performed using Tris-EDTA buffer (10 mM Tris base, 1 mM EDTA solution, pH 6.0) using a pressure cooker. Briefly, tissue sections were deparaffinized and rehydrated in graded alcohols, then incubated with blocking serum for 20 min. Following treatment with 3% H_2_O_2_ for 10 min to quench the endogenous peroxidase activity, sections were incubated with the primary antibody overnight at 4 °C. List of antibodies used: From Santa Cruz Biotechnology (Dallas, TX, USA): anti-LY75 (sc-515016 1:50) anti-vimentin (sc-373717, 1:200), anti-KLF4 (sc-166100, 1:40), anti-oct3/4 (sc-5279, 1:50), anti-Nanog (sc-376915, 1:50), anti-CD44 (sc-9960, 1:100), anti-ABCG2 (sc-37716,1:100),anti-Zeb1(sc-515793,1:100) anti-ALDH1A (sc-374149, 1:300), anti-EpCAM (sc-25308, 1:100), anti-Ovol2 (sc-515001, 1:50), anti-β-catenin (sc-133240, 1:100), anti-axin1 (sc293190, 1:100), anti-P53 (sc-126, 1:100) anti-RAD51 (sc-398587, 1:100) anti-MDM2 (sc-965, 1:100), anti-ki67 ( sc-23900 1: 100), from Millipore Sigma, ON, Canada: anti-γ-H2AX (07-627, 1:100). (From Abcam (Toronto, ON, Canada): anti-N-cadherin (ab-18203, 1:100), anti-E-cadherin (ab-1416, 1:100), anti-APC (ab40778, 1:200), anti-PUMA (ab-9643 1:100), from GeneTex: anti-GRHL2 (gtx109410, 1:150).

### 4.6. Immunofluorescence (IF)

IF analysis was performed as previously described [26]. Fixed samples were incubated with different primary antibodies, including anti-β-catenin (Santa-Cruz Biotechnology Dallas, TX, USA), anti-LY75 (Santa-Cruz Biotechnology Dallas, TX, USA and Abcam, Branford, CT, USA), anti-APC2 (Abcam, Branford, CT, USA), anti-Axin1 (Santa-Cruz Biotechnology Dallas, TX, USA), anti-N-cadherin, anti-E-cadherin, anti-vimentin, anti-EpCAM, and subsequently incubated with secondary antibodies, including rhodamine-linked goat-anti-mouse IgG1 (Santa Cruz Biotechnology Dallas, TX, USA) or Alexa Fluor 488-labeled goat anti-rabbit antibody (Abcam, Branford, CT, USA).

### 4.7. Western Blot

Western blot analyses for cells and tumor tissue fractions were performed as previously described [63]. The following antibodies were used for monitoring protein expression: from Santa Cruz Biotechnology (Dallas, TX, USA): anti-LY75 (sc-515016 1:500) anti-vimentin (sc-373717, 1:1000), anti-KLF4 (sc-166100, 1:500), anti-oct3/4 (sc-5279, 1:500), anti-Nanog (sc-376915, 1:500), anti-CD44 (sc-9960, 1:500), anti-ABCG2 (sc-37716,1:1000), anti-ALDH1A (sc-374149, 1:1000), anti-Bmi-1(sc-390443, 1:500), anti-EpCAM (sc-25308, 1:1000), anti-Ovol2 (sc-515001, 1:500), anti-B-catenin (sc-133240, 1:800), anti-axin1 (sc293190, 1:500), anti-β-Actin (sc-517582, 1:2000). From Abcam (Toronto, ON, Canada): anti-N-cadherin (ab-18203, 1:1000), anti-E-cadherin (ab-1416, 1:1000), anti-APC (ab40778, 1:1000), goat-anti-rabbit HRP conjugated (ab6721, 1:3000), goat-anti-mouse HRP conjugated (ab6789, 1:3000) From GeneTex (Hsinchu City, Taiwan): anti-GRHL2 (gtx109410, 1:1000). The same antibodies were used both for IHC and Western blot analyses.

### 4.8. Quantitative PCR (qPCR)

Quantitative PCR was performed as previously described [26]. Relative quantification of RNA expression was calculated using the 2^−ΔΔCq^ method [64]. The 18S ribosomal gene was used as an internal standard. Each sample was tested in triplicate. Primers were designed as previously shown [26]; all primers for qPCR are listed in Table A1.

## Figures and Tables

**Table 1:** Survival time of mice included in phase 1 experimental groups, and sites of metastasis formation.

Cells Injected IB	Animal ID (#)	Survival Days	Metastasis Sites
**SKOV3-M (shControl)**	14	243	Tumor nodules in gut mesentery Tumor nodules in pancreas/omentum Tumor nodules attached to dorsal wall
	15	148	
	16	201	
	17	140	
	18	243	
**Median survival**		**201**	
**SKOV3-E (shSKOV3-LY75-KD)**	20	78	Tumor in gut mesentery Tumor nodules in pancreas/omentun Tumor nodules attached to dorsal wall
	21	68	
	22	74	
	23	74	
**Median survival**		**74**	
**SKOV3-M/SKOV3-E (mixed; SKOV3-E+M)**	24	92	Small nodules in mesentery Tumor nodules in pancreas/omentum Invasion of liver Nodules attached to dorsal wall
	25	68	
	27	68	
	28	68	
	29	74	
**Median survival**		**68**	

## Abbreviations

EOC	Epithelial ovarian cancer
EMT	Epithelial mesenchymal transition
MET	Mesenchymal epithelial transition
EMP	Epithelial mesenchymal plasticity
SCID mice	Severe combined immunodeficient mice
IB injection	Intra-bursal injection
CSCs	Cancer stem cells
IHC	Immunohistochemistry
IF	Immunofluorescence
qPCR	Quantitative PCR

## Appendix A

**Table A1:** Primers used for quantitative PCR (qPCR).

Gene	q-PCR Primers	Sequence (5′-3′)
ZEB1 (NM_001128128.3)	Forward	CATTTCAGGGAAGCCTGGGT
	Reverse	CACCCTGTTAGGCAGTGAGG
	Product lengths (pb)	158
NANOG (NM_024865.4)	Forward	ATATTGCATGCCTCCTGGGG
	Reverse	GTGCCGGAAGCTTTTGTCTG
	Product lengths (pb)	104
POU5F1(Oct4) (NM_001285986.1)	Forward	AGATGCTTTGAGCTCCCTCTG
	Reverse	TTGGCTGAATACCTTCCCTGG
	Product lengths (pb)	124
CD44 (NM_001001391.2)	Forward	TCCCTGCTACCAGAGACCAA
	Reverse	GCTCCACCTTCTTGACTCCC
	Product lengths (pb)	106
CD133 (Prom1) (NM_001371406.1)	Forward	CCCCGCAGGAGTGAATCTTT
	Reverse	GAAGGACTCGTTGCTGGTGA
	Product lengths (pb)	137
ABCG2 (NM_004827.3)	Forward	GAGTTAACTGAGCTGGGGCA
	Reverse	TGACAGAATGTCAGGGCACC
	Product lengths (pb)	98
BMI1 (NM_005180.9)	Forward	TGCTTTGGTCGAACTTGGTG
	Reverse	TGCAGACTGGGGACAATGAA
	Product lengths (pb)	129
KLF4 (NM_001314052.2)	Forward	CCAAAAATGCGACCGAGCAT
	Reverse	TGTAGTGCTTTCTGGCTGGG
	Product lengths (pb)	174
ALDH1A1 (NM_000689.5)	Forward	TTGGACCAGTGCAGCAAATC
	Reverse	CGCCATAGCAATTCACCCAC
	Product lengths (pb)	171
